# Blood platelet factor 4: the elixir of brain rejuvenation

**DOI:** 10.1186/s13024-023-00681-w

**Published:** 2024-01-07

**Authors:** José M. Izquierdo

**Affiliations:** Centro de Biología Molecular Severo Ochoa. Consejo Superior de Investigaciones Científicas, Universidad Autónoma de Madrid (CSIC/UAM), C/ Nicolás Cabrera 1, Campus de Cantoblanco, Madrid, 28049 Spain

**Keywords:** Platelet factor 4, Neuroinflammation, Brain rejuvenation

Aging is invariably associated with some form of cognitive impairment. Three recently published articles in *Nature* family journals from independent groups reported that the plasma levels of platelet factor four (PF4) are negatively associated with brain aging and neurodegeneration phenotypes and positively associated with cognitive performance, brain rejuvenation and health. These studies identify a humble blood-derived chemokine as a potential elixir of brain youth.

**Fig. 1:**
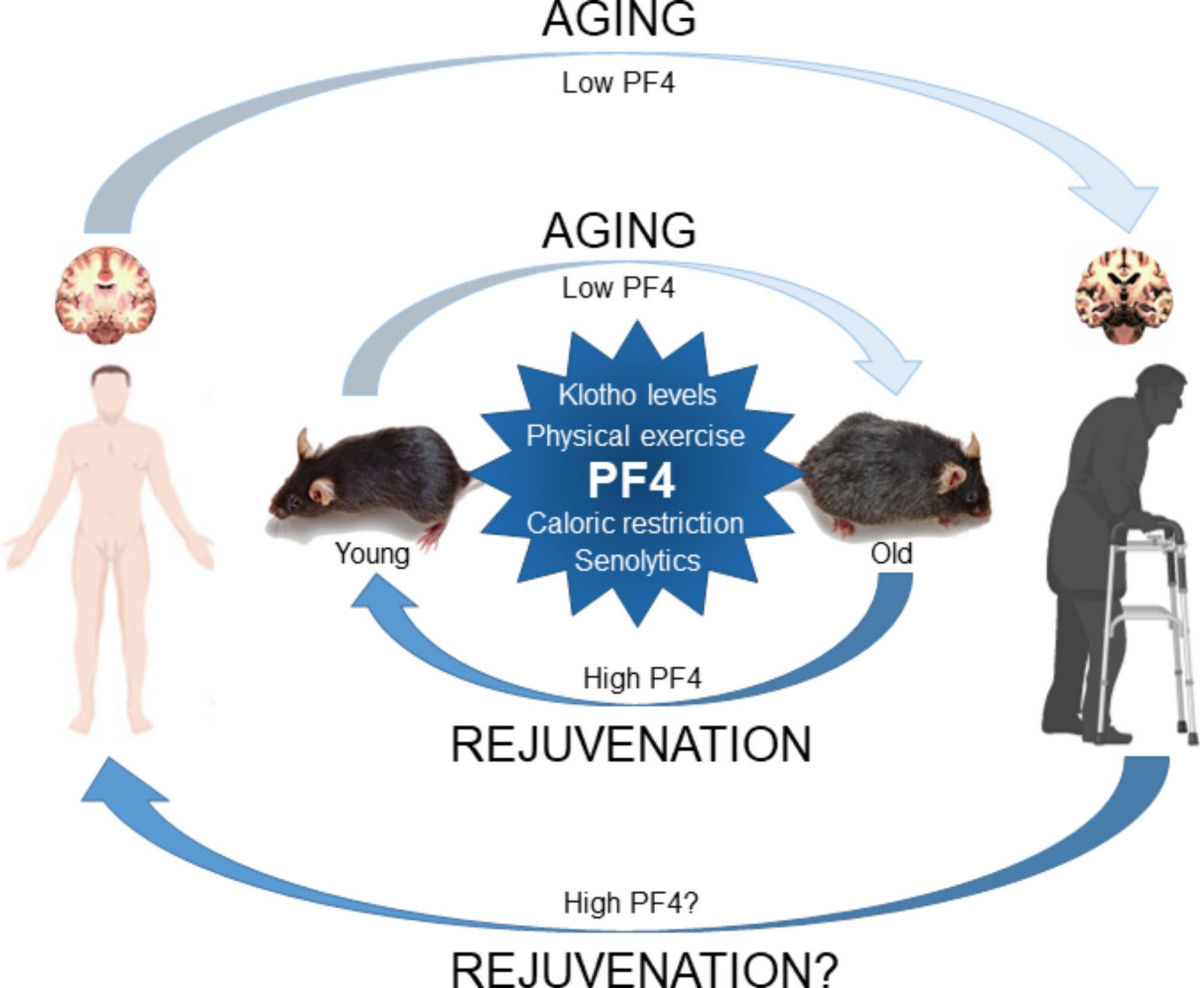
Overview of brain aging and rejuvenation in mice in a PF4-dependent manner. Reduced or increased levels of PF4 (blood platelet factor 4) lead to aging-associated neurodegeneration and brain rejuvenation in mice and potentially in humans, respectively. Administration of klotho, physical exercise, caloric restriction and/or senolytics increase PF4 levels, with beneficial effects on aging-associated neurodegeneration and brain rejuvenation

One of the three groups, led by Saul Villeda at the University of California at San Francisco (UCSF), had previously shown that administration of blood plasma from young mice rejuvenated the brains of old mice [1]. When they analyzed how young plasma differed from old plasma, they identified PF4 as the chemokine that transfers the restorative effects of young blood to aging brains [2]. Specifically, PF4 attenuated age-related neuroinflammation by rejuvenating the immune system, rescuing synaptic plasticity and improving hippocampal-dependent learning and memory in a partially CXCR3 chemokine receptor-dependent manner (Fig. 1). A second UCSF team led by Dena Dubal previously showed that klotho, a hormone linked to longevity, could improve cognition when administered to mice [3]; however, because klotho is too large to cross the blood-brain barrier, they concluded that the hormone must act indirectly in the brain. To look for this intermediary, they administered klotho to mice and measured changes in proteins in blood, finding that the levels of several platelet factors, including PF4, increased [4]. The Dubal team also found that systemic administration of PF4 improved neuronal connections in the hippocampus. Nevertheless, klotho effects were still observed in mice lacking PF4, suggesting that PF4 is sufficient but not necessary to recapitulate klotho-mediated cognitive enhancement and that there may be other unidentified platelet factors affecting cognition [4]. In the same vein, there are divergent mechanistic aspects between the observations of Shroer et al. [2], who suggested that the effects of PF4 occur in part through indirect immune mechanisms, and Park et al. [4], who suggested a more direct mechanism of action in the brain. Taken together, these findings may indirectly indicate the coexistence of multiple downstream mechanisms of action involving PF4-related factors, signaling cascades, immune responses, and/or cellular diversity/heterogeneity. Therefore, the mechanistic details and the inter-/intra-relationships between them should be investigated. On the other hand, the same team published a separate study in *Nature Aging* in July showing that klotho administration improved cognition in aging monkeys [5], but it is unknown whether the improvements involved PF4 (Fig. 1). In the third study, Tara Walker and her team at the University of Queensland (Australia) reported that platelet PF4 levels can be increased by exercise, and that administering PF4 directly into the brains of mice stimulated neurogenesis in the hippocampus, a critical region for memory [6]. They also observed that increasing systemic PF4 levels ameliorated age-related cognitive and regenerative impairment in a hippocampal neurogenesis-dependent manner, leading them to conclude that platelets play a major role in the rejuvenation of the aging brain (hippocampal learning and memory) associated with physical exercise during physiological aging. Collectively, the three studies show that PF4 improves cognition in aged mice [2, 4, 6] (Fig. 1).

A major limitation of these types of study is that few observations in mice translate into safe and effective therapies in humans. However, PF4 levels were found to be elevated in both young humans and mice compared with aged counterparts [2] and administration of human PF4 also improved cognition in mice [6]. Several “treatments” for brain aging are already available, such as physical exercise and calorie restriction, but they are often not an option for those most in need. Indeed, the Walker group demonstrated that platelets release PF4 into circulation after exercise [4], but many people with health conditions or advanced age are unable to exercise, so a pharmacological intervention could fill this gap. For example, platelets could be used to promote neurogenesis and counteract age-related cognitive decline [6]. Although more research is needed to understand how these activities rejuvenate the brain and to identify molecules that mimic their effects, it is possible that PF4-based treatments will be tested in clinical trials in the coming years. Future studies will also need to determine exactly how PF4 works in the body and brain, and whether it should ultimately form part of a therapeutic cocktail. These results might herald a new generation of drugs to dampen age-associated inflammatory and immunosenescent cellular phenotypes, such as taurine, metformin, rapamycin, NAD + precursors, sirtuin activators and antioxidants [7] (Fig. 1).

Platelets are principally known for their role in blood clotting and wound healing, and release platelet factors into the blood. The chemokine PF4 (also called anti-heparin factor or CXCL4) is found in the alpha granules of platelets and has a high affinity for heparin, and typically forms complexes with glycoproteins, such as protein C. In addition to coordinating the movement of leukocytes in inflammatory situations, chemokines are involved in myriad physiological and pathological processes including immune system development; immune surveillance, memory, response and regulation; inflammation; embryogenesis, angiogenesis and organogenesis; nervous system development and function; germ cell migration; cancer development and metastasis [6]. In this regard, the administration of PF4 may cause undesirable side effects. The most common complication of heparin treatment in surgical patients is bleeding, but the most dangerous complication is the development of heparin-induced thrombocytopenia (HIT) [8, 9], and all patients treated with heparin of any type and at any dose are at risk of HIT due to the formation of antibodies against the heparin-PF4 complex, which secondarily activates platelets and coagulation and ultimately leading to increased thrombin generation. The main manifestation is a 50% decrease in platelet count from baseline, and/or thrombotic complications occurring 5–14 days after initiation of heparin treatment [9]. This rare medical condition is also observed in a number of patients receiving the Oxford/AstraZeneca ChAdOx1-S vaccine against COVID-19 [8, 9]. Accordingly, the direct use of PF4 may not be free of risks *a priori*, such as those associated with the pathogenesis of different diseases including those related to infections (sepsis and viral infections), to tumors, as well as inflammatory processes, autoimmunity and cardiovascular, hepatic and renal diseases [6]. Therefore a deeper understanding of the molecular-mechanistic features of PF4 and its regulatory nodes during blood coagulation is necessary, which should occur hand-in-hand with the development of mimetic drugs to emulate the function and biological activity of this molecule in the absence of complications, favoring the maintenance and/or recovery of compromised brain capacity during aging, as well as during the development of neurological pathologies, dementias and even age-associated muscular dysfunction.

In summary, while PF4 may hold promise, it is perhaps only a small piece of a larger puzzle. For example, Katsimpardi et al. identified GDF11, a protein with comparable restorative effects that are associated with caloric restriction in aged mice [10]. The precise role played by these proteins remains unclear; however, if the present results in mice are reproduced in humans, it may be possible to reverse the effects of brain aging with a “brain rejuvenation elixir” found within the platelets of our blood. The explanation of why physical exercise, the longevity hormone “klotho” and blood transfusion from “young” individuals can induce cognitive improvement is linked to the action of a common factor––the secretion of PF4. The three-way carom suggests that the “holy grail” of brain rejuvenation might lie in understanding the role of PF4 in neuronal plasticity and organ homeostasis.
